# Supplementary material for: Comprehensive Annotation and Functional Exploration of MicroRNAs in Lettuce
Source: Front Plant Sci. 2021 Dec 24;12:781836. doi: 10.3389/fpls.2021.781836 (PMC8739914; doi:10.3389/fpls.2021.781836)
Supplement: Supplementary file 2 [file Data_Sheet_8.ZIP › GSM2230747.plot/Lsa-miRN1688_Lsat_1_v5_gn_6_30521.1_623_TPlot.pdf]

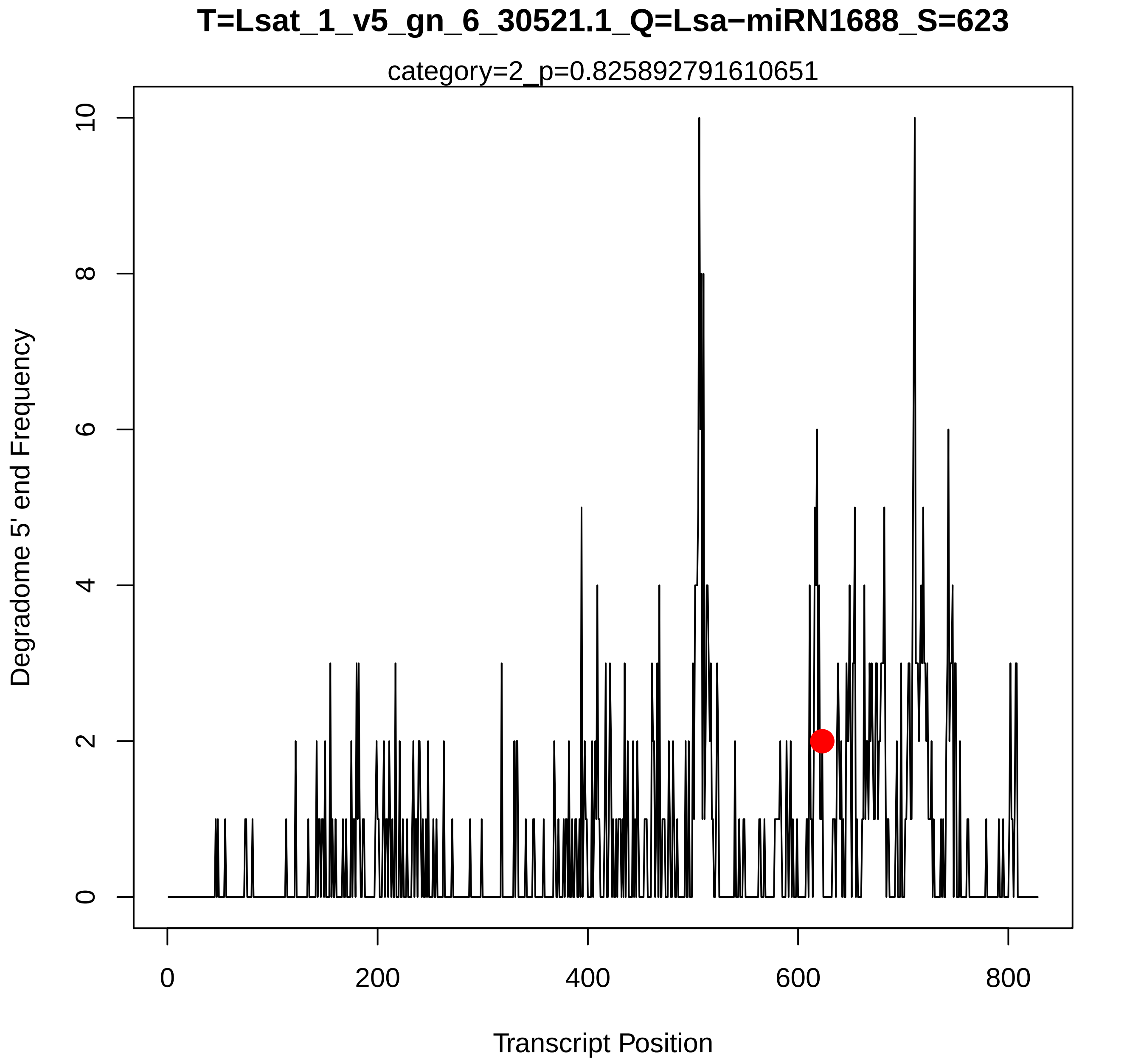
T=Lsat_1_v5_gn_6_30521.1_Q=Lsa−miRN1688_S=623
category=2_p=0.825892791610651
Degradome 5' end Frequency
Transcript Position
0
2
4
6
8
10
0
200
400
600
800
